# Cylinder Filling

**Published:** 1876-06

**Authors:** C. W. Spalding


### ARTICLE V.

### *Cylinder Fillings.*

BY C. W. SPALDING, D. D. S.

Of all the older methods of manipulating foil in the filling of teeth, that known as the cylinder method appears to be the one most likely to survive, and to successfully compete with those of more recent date. And why is it that this method bids fair to outlive the others? The answer is plain. It is because it possesses merits not possessed by either of the other older methods. One of the merits claimed for the cylinder method is that of great durability, especially in grinding surfaces, where the attrition of mastication subjects our operations to the test of both density and thoroughness. The writer has in his possession specimens of this kind of filling that have endured the wear and tear of from 25 to 35 years of hard service, and are still sound and perfect, and apparently would have lasted for another equally long period of time. Another meritorious point is, that this method necessitates a more thorough and consequently a better preparation of cavities preparatory to receiving the gold than is usually practiced. If this latter claim is well founded, the point is one of too great importance to be overlooked ; for I think it will be pretty generally conceded that the more modern methods tend to a little remissness in this particular A third claim is the comparatively greater facility with which any given cavity can be filled. Speed is often an important consideration, not only to the operator but also to the subject who is being operated upon. The adjustment of the rubber-dam (which is a very necessary safeguard for prolonged operations) frequently consumes as much time as would suffice to introduce a cylinder filling, and secure it against damage from moisture.

I merely state these claims without stopping to discuss them at this time, and pass at once to a description of the cylinder method as practised by the writer for many years.

In the preparation of the cylinders, layers of foil are smoothly laid one upon another, by rolling, until a sufficient diameter is obtained. It will scarcely be disputed that foil in smooth layers is more easily condensed than it is when worked in angular and irregular folds. Having the cylinders prepared of various lengths and diameters, they are placed in the cavity, nearly perpendicular to its floor, or if the floor is inclined then perpendicular to what will be the face of the plug when the operation is finished. The first cylinder introduced should, when practicable, be large enough to be easily kept in position. It should be as large as the cavity will receive without bruising or otherwise disturbing the form of the cylinder and disarranging the layers of which it is composed. This should now be condensed moderately against the wall of the cavity by means of any instrument having a suitable beak or foot. Very commonly one or two smaller cylinders must be introduced before the mass will firmly retain its place. But as soon as this is accomplished the mass must be thoroughly condensed before proceeding farther ; for if any defect is allowed at this stage of the operation, it will be very difficult to remedy it afterwards. Cylinder after cylinder is now introduced, until the cavity is nearly full; when, if the cavity is in the grinding surface, a cylinder may be placed against the walls on the side opposite to the point of beginning, and condensed there, and the finishing pieces inserted behind it. But in approximal cavities the cylinders on either side of the cavity should be so placed as to meet each other at the edge, and thus afford a thin protection to the margin of the cavity; and the last piece should be introduced between these two. Indeed the greatest difficulty encountered in the use of cylinders is in the introduction of the finishing pie es in approximal cavities. In all cases the finishing pieces should be tapering and rolled hard. They should also be chosen of a size agreeing with the size of the point of the condensing instrument, or as large as can be introduced without clogging, before the bottom of the opening is reached. The finishing pieces should never be very long and if the cavity is deep, foil, in some convenient form, should first be driven to the bottom of the cavity, filling the open space part way up, at the same time insuring close packing at the bottom of the cavity. Having thus lessened the depth of the opening, the hard-rolled tapering pieces are readily placed in position, and the whole is ready for the condensing of the surface. The cylinders having been chosen of a length sufficient to project somewhat beyond the margin of the cavity, are now to be condensed obliquely towards the centre, around the edge; and lastly, in a perpendicular direction. If all parts of the process have been carefully performed, the result will show a hard solid plug, capable of receiving a fine polish, and it is believed possessing greater density than is obtainable in any other way. In the one quality of durability in grinding surfaces, this property becomes manifest; and in this regard, the cylinder method may safely challenge comparison with any and every other, either new or old.

To prepare the cylinders cut a sheet of No. 4 foil into three, four, six, or eight strips, of equal width. Place one of these strips on a piece of cotton velvet, and press it at the middle with the thin sharp edge of a common ivory paper folder. If the edge is too thick reduce it. Now smooth the folded process until your fold is sufficiently narrow. The folding should be done *more* than four times, thus making the narrow fold or ribbon to consist of sixteen layers, and being of a length equal to the width of the sheet of foil. More folding makes the ribbon too stiff to wind smoothly. The length ot the cylinder is determined by the width of the ribbon, and consequently the length of the cylinder can be easily regulated by varying the width of the strips of foil into which the sheet is cut. Occasionally a half sheet may be required for making a long cylinder. It will be found better to add a second ribbon when a very large cylinder is required rather than fold a whole sheet, lest the ribbon should be too thick and clumsy.

A most convenient implement for winding the cylinder is a common broach cut off within about one-fourth of an inch of the handle. For rolling large cylinders it may be used of full size; but for small ones, it should be reduced to a very small diameter, made tempering, terminate in a slightly blunt point, and be polished smooth.

An entire ribbon makes a large cylinder. Of medium size a ribbon will make two, three, or four. Of the small cylinders, which are made from the narrow ribbons, from six to ten in number are made from each ribbon.

In rolling the cylinders wind the ribbon snugly upon the broach letting the ribbon slip between the thumb and finger until the desired diameter is obtained. Then break the ribbon by closing the thumb and finger upon it, and commence anew.

At the first, cylinders were made of soft foil exclusively, none other being manufactured at that time. The annealing of the gold after it was rolled into cylinders, first suggested itself to the writer as an aid in building up contour fillings. The advantage gained by annealing led to the subsequent trial of cohesive foil (so called) and ultimately to the gradual and entire abandonment of soft foil. The cohesive foil, or that annealed by the manufacturer, after beating, is soft enough for any purpose when not *re*-heated after being rolled into cylinders. A little more dexterity and care are requisite in handling annealed foil, but one soon learns to manipulate it successfully, and a harder plug is produced then when soft foil is used; as is evident from the superior finish which the annealed plug is capable of receiving.

But cylinders made from annealed or cohesive foil may, in most instances, be re-heated to advantage; but it is not necessary to heat those of tapering form designed for finishing the plug.

For the purpose of annealing, I have found a small, spoon-shaped platina dish, either with or without a handle, to be a good thing. I usually heat until the cylinders adhere slightly to each other when moved from side to side in the dish. At this time they assume that deepened shade of color peculiar to newly-annealed gold. Heated to this point they may be allowed to cool and be used at convenience. They will now unite very readily with each other in the cavity; under either strong hand pressure or the force of the mallet.

To those who are accustomed to use soft-foil cylinders I have a word to say. Try heating your cylinders moderately; not enough to thoroughly anneal the metal, but just sufficiently to burn any combustible matter that may be on the surfaces of the foil. Place say 20 cylinders, more or less, on a spoon, and heat until the smoke, arising from the combustion of the organic matter, is seen to escape. This will be seen the more readily if the spoon is moved aside from the flame for a moment, at short intervals.

This "purifying by fire," even if no more heat is employed than is just sufficient to consume the soilings from the fingers, will be found to greatly aid the welding process, and the filling will be harder, and will take on a better finish than when the heating is omitted.—*Missouri Dental Journal.*

